# Characterization of the complete chloroplast genome of *Aster subulatus* Michx

**DOI:** 10.1080/23802359.2019.1710599

**Published:** 2020-01-14

**Authors:** Xiao-jing Hu

**Affiliations:** College of Agriculture, Guizhou University, Guiyang, PR China

**Keywords:** *Aster subulatus* Michx, chloroplast genome, Carduoideae, phylogenetic relationship analysis

## Abstract

In this study, the chloroplast genome of *Aster subulatus* Michx, an important Chinese herb medical plant, has been presented using BGISEQ-500 sequencing. The chloroplast genome is 153,318 bp in size and is constituted of a pair of inverted repeat regions of 24,927 bp, a small single-copy region of 18,226 bp, and a large single-copy region of 85,238 bp. Totally, 102 unique genes, including 78 protein-coding genes, 20 tRNAs, and 4 rRNAs, were identified and annotated in the chloroplast genome. Phylogenetic maximum likelihood analysis indicated that *A. subulatus* Michx is closest to *A. hersileoides*.

*Aster subulatus* Michx (syn. *Symphyotrichum subulatum* (Michx.) G.L.Nesom), belonging to genus *Aster*, subfamily Carduoideae of Compositae, is an annual herb native to North America. Nowadays, it is also widely distributed at hillsides, forest edges, and roadsides in Jiangsu, Zhejiang, Jiangxi, Hubei, Hunan, Sichuan, and Guizhou provinces in China, and is an important Chinese herb medicine plant used for the external treatment of eczema, sore swollen poison, etc. In this study, its chloroplast genome has been assembled and characterized using BGISEQ-500 sequencing data. Besides, its relationship with several plant species of subfamily Carduoideae was investigated.

The specimen sample of *A*. *subulatus* Michx was isolated from Teaching Experiment Farm of Guizhou University, Huaxi District, Guiyang, Guizhou Province, China (26°21′29″N; 106°41′01″E) and the sample was deposited at Guizhou University. Total genomic DNA was extracted from fresh leaves according to the method prescribed by Yang et al. (2019) and stored at the Guizhou University (No. JDC01). High-quality genomic DNA was then used for the shotgun library construction and BGISEQ-500 sequencing. About 1 Gbp data were generated, and the obtained high-quality reads were first aligned to the chloroplast genomes of *Sonchus webbii* (NC_042383.1), *S. canariensis* (NC_042381.1), *S. boulosii* (NC_042244.1), *S. acaulis* (MK033507.1), *Lactuca sativa* (AP007232.1), *L. sativa* (DQ383816.1), *Cichorium intybus* (MK569377.1), *Youngia denticulata* (NC_042149.1), *Taraxacum platycarpum* (NC_031395.1), *T. officinale* (NC_030772.1), *T. obtusifrons* (NC_031815.1), *T. mongolicum* (NC_031396.1), *T. kok-aghyz* (NC_032057.1), *T. brevicorniculatum* (NC_032056.1), *T. brevicorniculatum* (NC_032056.1), and *T. amplum* (NC_031816.1). Then, the chloroplast genome was assembled and annotated according to the method prescribed by Yang et al. (2019). The annotated chloroplast genome has been deposited in Genbank with the accession number MN541093.

The *A*. *subulatus* Michx chloroplast genome is 153,318 bp in size. It contains a pair of 24,927 bp long inverted repeat regions, each separating a small single-copy region of 18,226 bp and a large single-copy region of 85,238 bp. Totally, 102 unique genes have been identified and annotated, including 78 protein-coding genes, 20 tRNAs, and four rRNAs, in the chloroplast genome. Among these genes, six protein-coding genes (*ndhB*, *rpl1*, *rpl23*, *rps7*, *rps12* and *ycf2*), four rRNAs (*rrn4.5*, *rrn5*, *rrn16* and *rrn23*), and five tRNAs (*trnA*, *trnG*, *trnI*, *trnN* and *trnT*) occurred in two copies, three tRNAs (*trnR*, *trnS* and *trnV*) occurred in three copies, and two tRNAs (*trnL* and *trnM*) occurred in four copies. The overall nucleotide composition of the chloroplast genome is: 31.4% A, 31.6% T, 18.4% C, and 18.6% G, with the total GC content of 37.0%.

Chloroplast genomes of six plant species belonging to Carduoideae and two plant species from Cichorioideae (as outgroup) downloaded from GenBank together with the *A*. *subulatus* Michx chloroplast genome were firstly subjected to sequence alignment using HomBlocks pipeline (Bi et al. 2018) and then were used for maximum likelihood tree construction according to the method described by Zhang et al. (2019). Phylogenetic maximum likelihood analysis indicated that *A. subulatus* Michx is closest to *A. hersileoides* (Figure 1). The complete chloroplast genome of *A. subulatus* Michx would provide valuable genetic information for Carduoideae plant researches.

**Figure 1.**
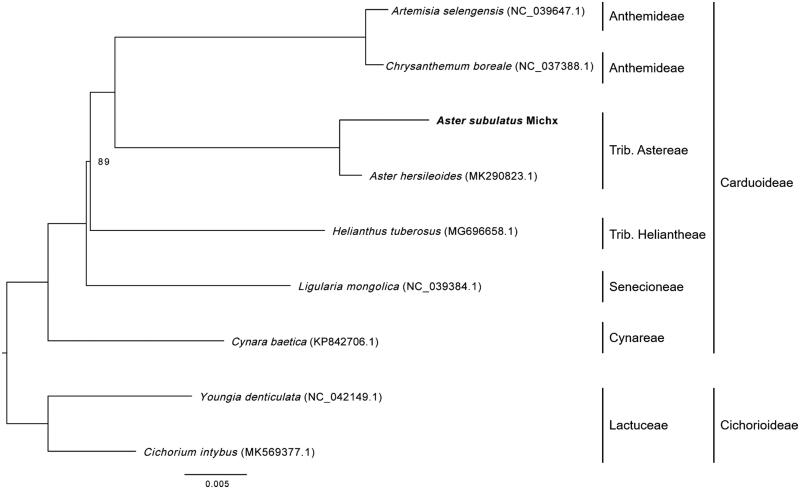
Maximum likelihood phylogenetic tree based on the complete chloroplast genome sequences of seven plant species from Carduoideae and two outgroup plant species from Cichorioideae.
